# Innovation through individual-focused transformational leadership in China: mediation by leader identification and cross-level moderation by team-focused leadership and innovation climate

**DOI:** 10.3389/fpsyg.2025.1615515

**Published:** 2025-07-30

**Authors:** Xiaokang Wang, Heetae Park, Hee Jin Kim

**Affiliations:** ^1^Shandong College of Economics and Business, Weifang, China; ^2^College of Business Administration, Dong-A University, Busan, Republic of Korea; ^3^College of Business, Gachon University, Seongnam, Republic of Korea

**Keywords:** individual-focused transformational leadership, team-focused transformational leadership, innovative behavior, leader identification, team innovative climate, cross-level analysis

## Abstract

In the new phase of economic transformation and social development, innovation has emerged as a critical issue. Corporate innovation largely depends on the innovative behavior of its members, making it crucial to understand the impact of transformational leadership on employees’ innovative behavior. We conducted a study from the perspective of relational identity theory. Questionnaire data were collected from April to June 2024 through a professional survey company in China that recruited 640 members and leaders of 160 teams. The analysis results are as follows: First, individual-focused transformational leadership was found to have a positive effect on individual innovative behavior. Second, leader identification played a significant mediating role in the relationship between individual-focused transformational leadership and individual innovative behavior. Third, team-focused transformational leadership exhibited a significant negative cross-level moderating effect on the relationship between individual-focused transformational leadership and leader identification. Lastly, team innovative climate showed a significant positive cross-level moderating effect on the relationship between leader identification and individual innovative behavior.

## 1 Introduction

Innovation is receiving unprecedented attention among Chinese enterprises (Shi, 2013). Innovation-driven companies like Alibaba, Huawei, and Tencent, which have achieved remarkable success in the global market, are inspiring others to follow their lead. This momentum is supported by China’s significant investment in research and development, which reached approximately 3.09 trillion yuan in 2023—a 10.4% increase from the previous year (China Daily, 2023; Xinhua News Agency, 2023), with 921,000 patents filed, the highest globally (Chen, 2024).

These achievements reflect China’s transition from the “world’s factory” to an innovation-oriented economy, positioning corporate innovation as a core national strategy (Xinhua News Agency, 2023). In today’s highly uncertain and complex environment, innovation plays a critical role in an organization’s survival and development (Xu and Yang, 2024). Since corporate innovation is primarily driven by the innovative behavior of its members, it is essential to explore the factors that promote employee innovative behavior (Amabile, 1988; Dai et al., 2022; Ud Din Khan et al., 2023). The antecedents of innovation are an important research topic that can help organizations survive and thrive in intense competition (Thao and Kang, 2018).

However, relying solely on members’ intrinsic motivation is insufficient; leadership plays a crucial role in stimulating and guiding these behaviors (Kang and Ham, 2021; Malibari and Bajaba, 2022; Shi et al., 2020). The current research particularly focuses on transformational leadership (TL). TL has gained prominence in leadership studies for its effectiveness in motivating employees to prioritize organizational goals over personal interests, thereby contributing to corporate development (Pillai and Williams, 2004; Walumbwa et al., 2005). Findings from empirical research show that TL significantly influences members’ behavior, attitudes, and performance (Bass et al., 2003; Greimel et al., 2023; Rafique et al., 2022; Sui et al., 2012).

To better understand the significance of TL in leadership studies in China, a keyword analysis of over 600 empirical research papers (2015–2023) from the China National Knowledge Infrastructure (CNKI) database was conducted using CiteSpace software. The results indicate the high centrality of transformational leadership, suggesting its widespread relevance and importance in shaping individual behaviors and organizational performance (see Table 1).

**Table 1:** CNKI literature database through keyword searches (count ≥ 50).

No.	Count	Centrality	Year	Keywords
1	93	0.36	2015 ~ 2023	Transformational leadership
2	71	0.24	2015 ~ 2023	Employee innovative behavior
3	51	0.14	2015 ~ 2023	Employee creativity
4	51	0.10	2015 ~ 2023	Innovative behavior
5	51	0.14	2015 ~ 2023	Servant leadership
6	50	0.09	2015 ~ 2023	Creativity

TL’s influence on innovation behavior has been widely studied (Li and Mao, 2018; Liu and Phillips, 2011; Pieterse et al., 2010; Zhu and Zhou, 2016). While much research has documented its positive impact, some scholars argue that TL can limit or even hinder individual innovation behavior (Basu and Green, 1997; Osborn and Marion, 2009; Tourish, 2013). Others have proposed that the relationship between TL and innovation may follow an inverted U-shaped curve, where the positive effects of TL diminish or even reverse beyond a certain point (Pang et al., 2019; Song et al., 2013). These conflicting results may arise from the multi-level nature of TL, which influences individuals and teams differently depending on the organizational and cultural context (Li et al., 2020).

To reflect the unique context of China, the distinction between individual-focused transformational leadership (ITFL) and team-focused transformational leadership (TTFL) (Wang and Howell, 2010) offers a useful framework. By contrast, contemporary Chinese views of cosmopolitanism are more collectivist and relational (Liu and Xie, 2023). Traditionally, Chinese culture has emphasized group goals, rooted in collectivist values such as harmony, relationships, and conformity (Wang et al., 2021). However, individualism is becoming increasingly important among younger generations, with uniqueness and diversity gaining value (Tolstikova et al., 2020). Additionally, the COVID-19 pandemic has disrupted traditional work structures. The shift to remote work and the growing need for individual autonomy have made individual-focused leadership more relevant, even in collectivist cultures (Greimel et al., 2023; Kniffin et al., 2021).

The current study examines the effects of TL on individual innovative behavior (IIB) in companies in China using individual-team cross-level approaches. Specifically, we explore the influence of ITFL on IIB while testing the moderating effects of team-level factors, such as team-focused leadership and team innovation climate (TIC). Moreover, ITFL fosters trust-based relationships that enhance leader identification (LI), thereby motivating members to engage in innovative behavior. By testing the mediating effect of LI, we seek to delineate the mechanisms through which leaders can effectively inspire individual innovation.

This research extends the field in at least three ways.

First, we distinguish and empirically examine the interaction between ITFL and TTFL. Although prior studies (e.g., Wang and Howell, 2010) have conceptually differentiated between individual-focused transformational leadership (ITFL) and team-focused transformational leadership (TTFL), our research goes further by empirically validating their interaction effects. Specifically, we find that TTFL has a negative cross-level moderating effect on the relationship between ITFL and leader identification (LI), revealing a potential competitive mechanism between the two leadership styles—an area that has not been fully explored in existing literature.

Second, we introduce and extend relational identity theory. While previous studies on transformational leadership have primarily focused on theoretical perspectives such as reciprocity (Wang and Howell, 2010), network theory (Cai et al., 2013), empowerment theory (Liu et al., 2019), and resource perspectives (Liang et al., 2018), this study innovatively applies relational identification theory (Sluss and Ashforth, 2007) as its theoretical framework. Rather than relying on traditional notions of “classical identification,” we emphasize relational identification to explain how ITFL enhances LI, which in turn fosters individual innovative behavior (IIB). This approach uncovers the psychological mechanisms through which leadership styles influence innovation.

Finally, we contribute through our cultural context and empirical design. This study analyzes multi-level, multi-source data collected from 640 employees nested within 160 teams in China, providing robust empirical support for the proposed theoretical model. Furthermore, by applying relational identification theory within the collectivist cultural context of China, we extend the cross-cultural applicability of the theory. This offers important theoretical implications for leadership research, which has predominantly been grounded in Western cultural settings.

## 2 Theoretical background and hypotheses

### 2.1 Multi-level TL

TL emphasizes not only the importance of teamwork, but also care for members and personal influence to nurture them (Park et al., 2023). It involves a range of behaviors that can be applied at the level of individual members or the collective team. Prior research has often overlooked this multi-level nature of TL, treating it as a holistic concept, which limits a comprehensive understanding of its effectiveness (Kim & Hong, 2016; Sun et al., 2016). From this perspective, a multi-level approach to the concept of TL has been proposed (Wang and Howell, 2010; Wu et al., 2010), suggesting that leaders exhibit two distinct leadership styles toward team members and the team: ITFL and TTFL.

ITFL includes individualized consideration and intellectual stimulation (Wang and Howell, 2010). The behavior of leaders with ITFL varies with different members. They strive to recognize the individual needs and capabilities of each member, allowing them to complete tasks according to their own work rhythms rather than viewing the team’s capabilities (Wang and Howell, 2010; Wu et al., 2010). In contrast, TTFL addresses all team members as a collective, influencing the group rather than focusing on individuals separately. The leadership style includes dimensions such as vision presentation and idealized influence, where leaders serve as exemplary role models for members to follow (Wang and Howell, 2010).

Despite the growing interest in multi-level TL, there are still a limited number of studies based on this approach (Feng, 2017; Wang and Howell, 2010), and only a few have distinguished the intrinsic levels of TL to explore their impact on individual innovative behavior (IIB) (Liang et al., 2018). Furthermore, to our knowledge, no prior studies have investigated the interaction between the two types of transformational leadership in the context of individuals’ innovation behavior. Therefore, the current study aims to differentiate the intrinsic levels of TL and examine their dynamics in relation to IIB.

To further ensure conceptual distinctiveness, we adopted validated constructs and made careful theoretical distinctions between ITFL and LI, while remaining vigilant about their potential overlap.

### 2.2 The relationship between ITFL and IIB

The relationship between ITFL and IIB can be explained by relational identity theory. According to this theory, members perceive themselves based on their relationship with their leader (Cooper, 2010; Sluss and Ashforth, 2007; Sluss et al., 2012). ITFL fosters close relationships with members through direct contact between leaders and members, allowing leaders to understand the uniqueness of each member and meet their psychological needs, which facilitates open communication (Wang et al., 2005). This individualized relationship encourages members to explore new ideas, take initiative, and present creative ideas without being constrained by organizational conventions.

Moreover, by emphasizing intellectual stimulation and the freedom to think independently, individual-focused transformational leaders help members recognize and identify their own strengths and roles within the team. This self-awareness promotes their ability to solve problems independently (Park and Yoon, 2015). Additionally, leaders who encourage autonomy provide support for independent decision-making, which enhances innovation (Gumusluoglu and Ilsev, 2009; Kanter, 1988). As a result, individuals begin to approach problems from multiple angles, generating new ideas and perspectives (Bass, 1985; Park and Kim, 2015). The following hypothesis is established:

*H1*: ITFL will have a positive effect on IIB

### 2.3 Mediating effect of LI

The attention and consideration provided by individual-focused transformational leaders cultivate close, direct, and unique relationships between members and their leaders. These connections are characterized by mutual trust, support, satisfaction, and personal attraction (Dumdum et al., 2013; Wang et al., 2005). The leader’s personal appeal fosters members’ identification with the leader (Kark and Shamir, 2002; Kark et al., 2003; Shamir et al., 1998), activating the relational self of subordinates and reinforcing LI (Kark and Shamir, 2002). Additionally, the individualized consideration inherent in ITFL provides support when members face difficulties (Carmeli et al., 2014), making them feel cared for by the leader. This personalized attention fulfills members’ socio-psychological needs, such as belonging, value, and identity, further strengthening LI.

Once LI is established, members begin to align their perspectives, goals, and interests with those of the leader and the organization (Van Knippenberg, 2000; Walumbwa and Hartnell, 2011). Transformational leaders encourage members to challenge conventional approaches and rethink established methods, stimulating innovation by drawing on their curiosity and imagination (Avolio et al., 1999). As LI strengthens, members become more intrinsically motivated to achieve organizational goals, feeling empowered to question existing norms and propose new ideas and approaches (Avolio et al., 1999). Furthermore, LI nurtures empathy, courage, and cooperation, inspiring members to participate more actively in innovative endeavors (Diah, 2014). The following hypothesis is established:

*H2*: LI will mediate the relationship between ITFL and IIB.

### 2.4 Cross-level moderating effect of TTFL

Unlike ITFL, TTFL focuses on aligning individual and collective goals and values (Wang and Howell, 2012). Leaders set a shared vision and benchmarks for the group. In response, individuals come to see their personal success as interconnected with the success of the team, perceiving their own goals and values as aligned with or dependent on the collective outcomes. This dynamic contributes to a heightened sense of self as part of the team (Turner et al., 1979), where individuals view themselves not just as independent entities but rather as members of a larger collective. Those with a strong sense of identification with the group feel a greater sense of belonging and responsibility toward the team, which often results in stronger cohesion and unity (Van Knippenberg et al., 2004).

While TTFL strengthens team unity, it can simultaneously limit individual innovation. For team-oriented actions, TTFL inherently requires fairness and equality, fostering trust and cohesion among members. However, this emphasis on equal treatment can lead employees to follow rigid routines, stifling their capacity for innovation (Song et al., 2013). While fairness is essential for team cohesion, it can unintentionally suppress individual creative behavior. Additionally, by setting high performance expectations and encouraging learning from role models, TTFL can inadvertently increase work pressure. This increased pressure may result in emotional exhaustion, which undermines trust in the leader and weakens the employee’s identification with the team, thus hindering the adoption of new knowledge and technologies (Cai et al., 2013). In this environment, employees may be less inclined to question established practices, build personal relationships with leaders, or experiment with new methods for completing tasks. As a result, TTFL may negatively moderate the relationship between ITFL and LI, thereby negatively affecting IIB. The following hypothesis is proposed:

*H3*: TTFL will moderate the relationship between ITFL and LI. Specifically, when TTFL is high, the relationship between ITFL and LI will weaken, and when it is low, the relationship will be strengthened.

### 2.5 Cross-level moderating effect of TIC

TIC is the shared perception of the work environment that influences team members’ ability to exercise their innovative capabilities (Jiang et al., 2023; Pfeffer and Salancik, 1977). It captures the broader, collective atmosphere that shapes how innovation is approached and valued by the group. Prior research suggests that this team-level variable plays a role as an important situational variable that affects individual members’ attitudes, motivation, and behaviors (Wang and Zhu, 2006).

Teams in a highly innovative climate exhibit a positively charged environment that fosters innovation by forming a strong psychological field that motivates both leaders and members to explore the unknown and try new methods (Lewin, 1943). When team atmosphere and norms emphasize creativity and innovation as core values, members are motivated to engage more directly and actively in creative activities (Jiang et al., 2023; West, 1990). This environment also fosters a cultural atmosphere in which taking risks and attempting innovative solutions in the face of challenges and obstacles is accepted (Baer and Oldham, 2006).

In addition, this environment recognizes and values innovative behavior across the team, facilitating interactions between leaders and members and encouraging new initiatives. This environment facilitates interaction between leaders and members, encouraging the pursuit of innovative actions. As members increasingly identify with their leader, they are more likely to be motivated to dedicate resources and energy to innovative efforts. Consequently, in such an environment, a strong positive relationship between LI and members’ innovative behavior is expected to emerge.

Conversely, in teams with a low innovative climate, the environment is dominated by traditional performance indicators such as efficiency and reliability. This environment creates a psychological field that suppresses innovation, pushing members to maintain the status quo and optimize existing procedures. The leader’s influence may not be sufficient to overcome the inertia of this environment, leading to decreased innovative behavior. In such a scenario, team norms and atmosphere do not particularly encourage innovation, and greater emphasis is placed on traditional performance metrics like efficiency and reliability (Hirst et al., 2009).

**Figure 1:**
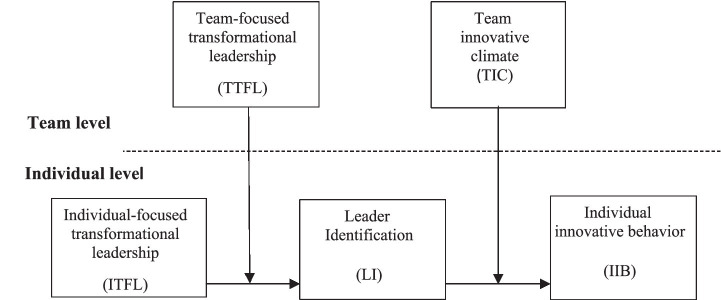
Research model.

In a low innovative climate or in situations where innovation is restricted, it may be difficult for LI to promote creative outcomes. Even if members are motivated by their loyalty and identification with the leader, their efforts are more likely to be directed toward optimizing existing practices or reducing waste to improve efficiency. While these activities benefit the organization commercially, they offer little creative value. In these teams, members may feel that creativity is neither necessary nor beneficial for achieving the leader’s goals, leading them to focus on activities that do not require high levels of innovation (Diah, 2014). As a result, the relationship between LI and innovation is likely to weaken. The following hypothesis is proposed (Figure 1):

*H4*: TIC will moderate the relationship between LI and IIB. Specifically, when TIC is high, the relationship between LI and IIB will be strengthened, and when it is low, the relationship will be weakened.

## 3 Methods

### 3.1 Participants and data collection

TIC is the participants in this study were leaders and members working in local enterprises in China, comprising individuals from various industries and occupations. To ensure the accuracy and authenticity of the measurements, this study adopted a matching collection method. Team members completed a questionnaire designed for members, while team leaders completed a questionnaire designed for leaders. Members reported on the transformational leadership style of their team leaders, LI, TIC, leader-member exchange (control variable), and personal demographic characteristics. The leader questionnaire was filled out by the direct leaders of the members, with questions assessing the innovative behaviors of the respective members.

Data collection was conducted by a professional survey company in China from April to June 2024. During this period, the research team maintained continuous monitoring and follow-up to ensure an adequate sample size and diversity of respondents. Upon completion, the questionnaires were retrieved via WeChat and email.

Data from one leader and four members from each team were collected, resulting in a total of 705 member responses and 179 leader responses. Following a rigorous screening process to eliminate insincere and incomplete questionnaires, the responses were matched on a one-to-one basis between team leaders and their respective team members. If any response from a team member or leader was incomplete the entire team’s data were excluded from the analysis. Data exclusions were based on the following predefined criteria to ensure data quality and validity: (1) responses with identical answers to all items (suggesting inattentive responding), (2) cases with missing data on key variables, and (3) responses completed in under 100 s (indicating insufficient attention). These criteria were established prior to data analysis. Ultimately, 160 teams and 640 questionnaires were used for analysis, yielding a pass rate of 89%. An *a priori* power analysis was conducted using G*Power 3.1. Assuming a medium effect size (*f*^2^ = 0.15), *α* = 0.05, and power (1 – *β*) = 0.90, the required sample size was calculated as 99. Accounting for a 5% attrition rate, the adjusted minimum sample size was set at 105. The final dataset includes 160 teams (Level-2 units), which exceeds the required sample size, thereby ensuring sufficient statistical power to detect medium-sized fixed effects.

### 3.2 Measures

All scales used in the survey items of this paper were adopted from previous studies. A 5-point Likert scale was used for measurement, with 1 indicating “strongly disagree” and 5 indicating “strongly agree” except for demographic variables.

### 3.3 Individual-focused transformational leadership

ITFL was measured using the eight questionnaire items developed by Zhang et al. (2013). This scale, originally developed by Podsakoff et al. (1990) and adapted for use in the Chinese context, was validated by Li and Liu (2014). ITFL consists of two dimensions: intellectual stimulation and individualized consideration. Sample items include: “My team leader considers my feelings when making decisions,” “My team leader motivates me to think about old problems in new ways”.

### 3.4 Team-focused transformational leadership

TTFL was measured using the 15 questionnaire items developed by Zhang et al. (2013), based on the study by Podsakoff et al. (1990) and adapted for use in the Chinese context. This scale was validated by Li and Liu (2014). TTFL comprises four dimensions: articulating a vision, providing an exemplary model, setting high-performance expectations, and promoting team cooperation. Sample items include: “Our team leader clearly understands the team’s goals,” “Our team leader articulates a vision necessary for our team”.

### 3.5 Individual innovative behavior

IIB was measured using the six questionnaire items developed by Scott and Bruce (1994), which was validated in the Chinese cultural context by Liu and Shi (2009) and Zhu et al. (2015). Sample items include: “Team member (#1 ~ 4) tries new techniques or methods when performing tasks,” “Team member (#1 ~ 4) often has creative ideas or new thoughts”.

### 3.6 Leader identification

This study references previous research methods (Kark et al., 2003; Walumbwa and Hartnell, 2011) and uses the organizational identification scale (Mael and Ashforth, 1992) adapted to measure LI. The word “organization” in the questionnaire was changed to “leader,” resulting in six items. Sample items include: “When someone criticizes my team leader, it feels like a personal insult,” “I care very much about how others perceive my team leader.”

### 3.7 Team innovation climate

TIC was measured using the 8-item scale adapted by Sui et al. (2012) from the questionnaire developed by Anderson and West (1998). Sample items include: “Our team is dedicated to pursuing innovation in our work,” “Our team always supports innovative ideas when they are proposed.”

### 3.8 Control variables

Demographic variables that are expected to influence the relationships between variables were controlled, including gender, marital status, education level, age, years of service, years of service with the current supervisor, and employment status. In addition to demographic variables, leader-member exchange (LMX) was used as a control variable at the individual level. Given that the quality of the leader-member exchange relationship is likely to affect members’ identification with their leader, LMX was included as a control variable. LMX was measured using the scale developed by Graen and Uhl-Bien (1995).

### 3.9 Analytical approach

To validate the legitimacy of the research model, confirmatory factor analysis (CFA) was carried out using AMOS 24. To test our research hypotheses at the individual level, SPSS 27 was employed. The mediating effect of LI was tested using PROCESS 3.3. A cross-level moderator model refers to a model in which two variables at different levels interact to predict outcomes at a lower level of analysis. ICC(1), ICC(2), and rWG are the most commonly used methods for verifying reliability in multilevel organizational research (James et al., 1984). Here, reliability refers to the degree of consistency in the responses of the respondents (Bliese, 2000; Kozlowski and Hattrup, 1992). Following the confirmation of reliability and validity, the cross-level effects between variables were analyzed. The cross-level moderating effects of TTFL and TIC were analyzed using HLM 6.08.

The respondents’ profiles are presented in Table 2.

**Table 2:** Demographics of respondents.

Demographics of respondents	Frequency	Percentage
Gender		
Male	355	55.5
Female	285	44.5
Level of education		
Under 20	5	0.8
20–29	303	47.3
30–39	285	44.5
40–49	40	6.3
50 and above	7	1.1
Education		
High school	77	12.0
Junior college	240	37.5
4-year university	299	46.7
Graduate school or higher	24	3.8
Employment status		
Regular	623	97.3
Non-regular	17	2.7
Experience (in Years)		
1 or less	115	18.0
2–5	339	53
6–9	177	27.7
10 of more	9	1.4
Working with current leader (in Years)		
1 or less	141	22.0
2–5	381	59.5
6–9	114	17.8
10 of more	4	0.6

### 3.10 Ethical considerations

This study was conducted in accordance with established ethical guidelines for research involving human participants. Prior to participation, all respondents were informed about the purpose of the study, the voluntary nature of their participation, and their right to withdraw at any time without penalty. Informed consent was obtained from all participants before data collection. No personally identifiable information was collected, and all responses were treated anonymously and confidentially.

## 4 Results

All anonymized data and study materials used in this research are publicly available via the Open Science Framework (OSF): https://osf.io/y68gp/. This ensures transparency and allows for independent verification and replication of the analyses.

### 4.1 Descriptive statistics and correlations

Table 3 presents the mean, standard deviation, correlation, and Cronbach’s alpha values. It also includes the Rwg and ICC values for team-level variables. The Cronbach’s alpha coefficients for each dimension of this scale are all above 0.86, indicating that all variables have high reliability. The results of the correlation analysis show significant correlations consistent with the hypotheses among the research variables, both at the individual and team levels.

**Table 3:** Reliability and correlation analysis results between individual and team-level variables.

Individual level 1	*M*	SD	1	2	3	4
1. LMX	3.59	0.97	(0.94)			
2. ITFL	3.49	0.78	0.42^**^	(0.86)		
3. LI	3.65	0.84	0.55^**^	0.52^**^	(0.89)	
4. IIB	3.25	1.00	0.49^**^	0.34^**^	0.50^**^	(0.90)

Team level 2	*M*	SD	ICC1	ICC2	r_WG_	1	2
1. TTFL	3.80	3.84	0.15	0.42	0.89	(0.88)	
2. TIC	3.84	0.83	0.60	0.86	0.84	0.17^*^	(0.93)

Level 1 N = 640, Level 2 N = 160, *p < 0.05, **p < 0.01, ***p < 0.001, the number in parentheses is the Cronbach’s Alpha. LMX, Leader-Member Exchange; ITFL, Individual-focused Transformational Leadership; LI, Leader Identification; IIB, Individual Innovative Behavior; TTFL, Team-focused Transformational Leadership; TIC, Team Innovative Climate; M, Mean; SD, standard Deviation; ICC, Intra-Class Correlation Coefficient; R_wg_ within group agreement.

### 4.2 Confirmatory factor analysis

To assess the construct validity of the research variables, CFA was performed for the individual and team levels. At the individual level, several indicators confirmed the model’s fit. The *χ*^2^/df value was 2.90, which is below the acceptable threshold of 3.00. The RMR value was 0.04, which is below the threshold of 0.08, indicating a high level of fit. The GFI value was 0.91, exceeding the preferred benchmark of 0.90, and the NFI value was also above the threshold at 0.92. The IFI and TLI values were 0.95 and 0.94, respectively, both exceeding the threshold of 0.90, and the CFI value was very high at 0.95. Finally, the RMSEA value was 0.06, which is below the threshold of 0.08, indicating a high level of model fit.

Regarding the potential issue of conceptual overlap between variables—particularly between ITFL and LI—which may lead to multicollinearity or inflated mediation effects, we conducted a multicollinearity diagnostic using Variance Inflation Factor (VIF) analysis during the preliminary stage. The results showed that all VIF values ranged from 1.018 to 4.899, which are below the commonly accepted threshold of 5. This indicates that there is no severe multicollinearity in the model.

At the team level, similar results were observed. The *χ*^2^/df value was 2.56, below the threshold of 3.00, indicating a good fit with the data. The RMR value was 0.04, below the threshold of 0.08, indicating a high level of model fit. The GFI value was 0.93, exceeding the threshold of 0.90, and the NFI value was also above the threshold at 0.93. The IFI and TLI values were both 0.95, exceeding the threshold of 0.90, and the CFI value was very high at 0.95. Finally, the RMSEA value was 0.05, which is below the threshold of 0.08, indicating a very high level of model fit.

At the individual level, given the high correlation between ITFL and LI, the fit of an alternative model was evaluated by combining ITFL and LI into a single factor to compare with the proposed factor structure. The analysis results were *χ*^2^/df = 6.98, RMR = 0.09, GFI = 0.74, NFI = 0.81, IFI = 0.83, TLI = 0.81, and CFI = 0.83, indicating that the four-factor model is more valid than the three-factor model. At both the individual and team levels, all variables satisfied the standard thresholds, with average variance extracted (AVE) values above 0.5 and composite reliability (CR) values greater than 0.7. Furthermore, all standardized factor loadings for the predictor variables were higher than 0.50.

The specific results are shown in Table 4.

**Table 4:** Results of confirmatory factor analysis at individual and team levels.

Individual-level variables		Factor loading	CR	AVE	Team-level variables		Factor loading	CR	AVE
ITFL	IC 1	0.75	0.87	0.62	TTFL	VP 1	0.74	0.84	0.52
	IC 2	0.81				VP 2	0.71		
	IC 3	0.83				VP 3	0.77		
	IC 4	0.74				VP 4	0.68		
	IS 5	0.71	0.82	0.54		VP 5	0.69		
	IS 6	0.72				EB 6	0.82	0.80	0.57
	IS 7	0.78				EB 7	0.66		
	IS 8	0.73							
LI	LI 1	0.75	0.89	0.57		EB 8	0.77		
	LI 2	0.71							
	LI 3	0.76				TCF 9	0.73	0.82	0.53
	LI 4	0.79							
	LI 5	0.72				TCF 10	0.74		
	LI 6	0.81							
IIB	IIB 1	0.83	0.95	0.71		TCF 11	0.69		
	IIB 2	0.84							
	IIB 3	0.83				TCF 12	0.75		
	IIB 4	0.85				HPE 13	0.80	0.80	0.57
	IIB 5	0.82				HPE 14	0.65		
	IIB 6	0.88				HPE 15	0.81		
LMX	LMX1	0.78	0.90	0.60	TIC	TIC 1	0.80	0.93	0.62
	LMX2	0.73				TIC 2	0.78		
	LMX3	0.79				TIC 3	0.80		
	LMX4	0.77				TIC 4	0.77		
	LMX5	0.77				TIC 5	0.75		
	LMX6	0.81				TIC 6	0.80		
	LMX7	0.85				TIC 7	0.80		
						TIC 8	0.80		

CR, Composite Reliability; AVE, Average Variance Extracted; ITFL, Individual-focused Transformational Leadership; IC, Individual Consideration; IS, Intellectual Stimulation; LI, Leader Identification; IIB, Individual Innovative Behavior; LMX, Leader-Member Exchange; TTFL, Team-focused Transformational Leadership; VP, Vision Presentation; EB, Exemplary Behavior; TCF, Team Cooperation Facilitation; HPE, High Performance Expectations; TIC, Team Innovative Climate.

### 4.3 Hypothesis testing

To test hypothesis 1, individual-level regression analysis was employed. As shown in Model 2 in Table 5, ITFL still has a significant positive impact on IIB even after controlling for the effect of demographic variables and LMX in Model 1 (*β* = 0.37, *t* = 9.95, *p* < 0.001). Thus, H1 is supported.

**Table 5:** Results of individual-level regression analysis.

Variable	IIB						LI			
	Model 1		Model 2		Model 3		Model 4		Model 5	
	*β*	*t*	*Β*	*t*	*Β*	*t*	*β*	*t*	*β*	*t*
Constant	1.64^***^	4.39	0.84^*^	2.35	0.56†	1.69	1.54^***^	4.18	0.70^*^	2.00
Gender	0.14^*^	2.38	0.08	1.50	0.04	0.74	0.17^**^	2.95	0.11^*^	2.01
Age	0.11	1.59	0.09	1.41	0.07	1.13	0.08	1.16	0.06	0.93
Education	0.00	0.02	−0.02	−0.50	−0.03	−0.86	0.05	1.20	0.03	0.74
Marital status	0.16^*^	2.11	0.13†	1.80	0.08	1.25	0.15†	1.98	0.12†	1.65
Employment status	−0.29	−1.61	−0.17	−1.01	−0.19	−1.20	−0.09	−0.49	0.04	0.23
Years of service	0.13	1.41	0.11	1.34	0.06	0.76	0.15	1.67	0.13	1.63
Years with current supervisor	−0.20^*^	−2.31	−0.16^*^	−2.03	−0.09	−1.25	−0.21^*^	−2.50	−0.17^*^	−2.23
LMX	0.46^***^	15.66	0.33^***^	11.22	0.21^***^	7.16	0.43^***^	14.96	0.30^***^	10.39
ITFL			0.37^***^	9.95	0.22^***^	5.74			0.39^***^	10.69
LI					0.40^***^	10.77				
*R* ^2^	0.31		0.40		0.49		0.29		0.40	
Δ*R*^2^	0.31		0.09		0.09		0.29		0.11	
ΔF	34.56^***^		99.01^***^		115.95^***^		31.96^***^		114.27^***^	

*N* = 640, †*p* < 0.1, *p < 0.05, **p < 0.01, ***p < 0.001. LMX, Leader-Member Exchange; ITFL, Individual-focused Transformational Leadership; LI, Leader Identification; IIB, Individual Innovative Behavior.

To test hypothesis 2, we employed both the Baron and Kenny (1986) method and the bootstrapping technique (Hayes, 2009). The results of the Baron and Kenny method are as follows: (1) In Model 2, the direct effect of ITFL on IIB shows a significant positive impact (*β* = 0.37, *p* < 0.001). (2) In Model 4, the effect of ITFL on LI shows a significant positive impact (*β* = 0.39, *p* < 0.001). (3) In Model 3, with LI added as a mediating variable, the direct effect of ITFL on IIB weakens (*β* = 0.22, *p* < 0.001). At the same time, the effect of LI on IIB is significant (*β* = 0.40, *p* < 0.001), confirming the partial mediating role of LI. Bootstrapping analysis (Hayes, 2009) produced similar results, showing a significant mediation effect of LI with a 95% confidence interval [indirect effect = 0.30, 95% CI = (0.23, 0.37)], which did not include 0, thereby supporting hypothesis 2.

To test the cross-level moderation effect of TTFL (hypothesis 3), in Model 1, gender, age, education, marital status, employment status, years of service, years of service with the current supervisor, and LMX were included as Level-1 control variables. ITFL was included as the Level-1 independent variable, and the interaction term between ITFL and TTFL was included as the Level-2 independent variable. LI was used as the dependent variable to construct the regression model. The results of the analysis using the cross-level model are as follows:

Level 1 Model:

$$\begin{array}{l} (\mathrm{LI})\mathrm{ij}=\beta \mathrm{0j}+\beta 1j^{*}\;(\text{Genderij})+\beta 2j^{*}\;(\text{Ageij})+\beta 3j^{*}\;(\text{Educationij})+\\ \beta 4j^{*}\;(\text{Marital Statusij})+\beta 5j^{*}\;(\text{Employment Statusij})+\\ \beta 6j^{*}\;(\text{Years of Serviceij})+\beta 7j^{*}\;(\begin{array}{l} \text{Years with Current}\;\\ \text{Supervisorij} \end{array})+\\ \beta 8j^{*}\;(\text{LMXij})+\beta 9j^{*}\;(\text{ITFLij})+\mathrm{rij} \end{array}$$

Level 2 Model:

$$\beta 0j=\gamma 00+\gamma 01^{*}\;(\text{TTFLj})+\mu 0j$$

$$\begin{array}{l} \beta 1j=\gamma 10,\beta 2j=\gamma 20,\beta 3j=\gamma 30,\beta 4j=\gamma 40,\\ \beta 5j=\gamma 50,\beta 6j=\gamma 60,\beta 7j=\gamma 70,\beta 8j=\gamma 80,\\ \beta 9j=\gamma 90+\gamma 91^{*}\;(\text{TTFLj}) \end{array}$$

Mixed Model:

$$\begin{array}{l} (\mathrm{LI})\mathrm{ij}=\gamma 00+\gamma 01^{*}\;(\text{TTFLj})+\gamma 10^{*}\;(\text{Genderij})+\gamma 20^{*}\;(\text{Ageij})+\\ \gamma 30^{*}\;(\text{Educationij})+\gamma 40^{*}\;(\text{Marital Statusij})+\\ \gamma 50^{*}\;(\text{Employment Statusij})+\gamma 60^{*}\;(\begin{array}{l} \text{Years of}\;\\ \text{Serviceij} \end{array})+\\ \gamma 70^{*}\;(\text{Years with Current Supervisorij})+\\ \gamma 80^{*}\;(\text{LMXij})+\gamma 90^{*}\;(\text{ITFLij})+\\ \gamma 91^{*}\;{\left(\text{TTFLj}\right)}^{*}\;(\text{ITFLij})+\mu 0j+\mathrm{rij} \end{array}$$

The cross-level analysis is divided into Models 1 through 4 to clearly illustrate the impact of each factor. In Model 1, gender, age, education, marital status, and other Level-1 variables are controlled, revealing a strong positive effect of LMX on LI (*β* = 0.70, *p* < 0.001). In Model 2, ITFL is added, showing a significant positive impact on LI (*β* = 0.67, *p* < 0.001) and indicating that ITFL positively influences LI. Model 3 tests the direct effect of TTFL on LI (*β* = 0.11, *p* < 0.05); there is a positive influence, though not as strong as ITFL. In Model 4, the interaction between ITFL and TTFL is introduced, and the results show a significant negative moderating effect (*β* = −0.41, *p* < 0.001), suggesting that when TTFL is low, the effect of ITFL on LI is stronger. The ICC value of the model is 0.33, indicating that 33% of the variance within the model is due to team-level differences, supporting the appropriateness of cross-level analysis. The residual variance (σ^2^) and the variance of the random intercept (*τ*) also support the model’s fit. Therefore, hypothesis 3 is supported. The specific results are shown in Table 6.

**Table 6:** Results of cross-level hierarchical regression analysis (hypothesis 3).

Variable	LI							
	Model 1		Model 2		Model 3		Model 4	
	*β*	*t*	*β*	*t*	*β*	*t*	*β*	*t*
Constant *γ*_00_	3.26^***^	17.42	3.37^***^	20.83	3.34^***^	20.68	3.52^***^	22.92
Gender *γ*_10_	0.01	0.35	0.01	0.29	0.01	0.31	0.01	0.33
Age *γ*_20_	0.09^*^	2.47	0.06†	1.77	0.06†	1.84	0.05†	1.71
Education *γ*_30_	0.02	0.91	0.01	0.36	0.00	0.22	−0.00	−0.23
Marital Status *γ*_40_	0.11^**^	2.83	0.10^**^	2.88	0.10^**^	2.98	0.09^**^	2.80
Employment Status *γ*_50_	0.01	0.10	0.04	0.53	0.05	0.66	0.04	0.47
Years of Service *γ*_60_	−0.03	−0.03	−0.83	1.34	−0.03	−0.82	−0.03	−0.78
Years with Current Supervisor *γ*_70_	0.02	0.02	0.42	−2.03	0.02	0.61	0.02	0.52
LMX *γ*_80_	0.70^***^	24.97	0.30^***^	8.83	0.24^***^	5.94	0.23^***^	5.96
ITFL *γ*_90_			0.67^***^	16.78	0.66^***^	16.51	0.41^***^	8.60
TTFL *γ*_01_					0.11^*^	2.29	0.02	0.49
ITFL * TTFL γ_91_							−0.41^***^	−8.96
σ^2^	0.09	0.08	0.07	0.07				
τ	0.10		0.03		0.04		0.03	
ICC	0.52		0.31		0.33		0.33	

Level 1 N = 640, Level 2 N = 160, †*p* < 0.1, *p < 0.05, **p < 0.01, ***p < 0.001 LMX, Leader-Member Exchange; ITFL, Individual-focused Transformational Leadership; TTFL, Team-focused Transformational Leadership; LI, Leader Identification; σ^2^, Residual Variance; τ, Random Intercept; ICC, Variance Within the Model.

**Figure 2:**
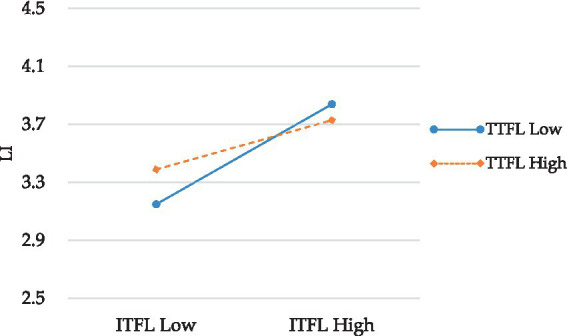
Interaction Plot of TTFL and ITFL.

To understand the nature of moderation, a simple slope test was conducted. The results, depicted in Figure 2, show that when TTFL is low, the relationship between ITFL and LI is strong (−1 SD; *β* = 0.41, *p* < 0.01), but when TTFL is high, the relationship weakens (+1 SD; β = 0.01, ns). This pattern of results supports hypothesis 3.

To test the cross-level moderation effect of TIC (hypothesis 4), in Model 2 of Table 6, gender, age, education, marital status, employment status, years of service, years with the current supervisor, and LMX were included as Level-1 control variables. LI was included as the Level-1 independent variable, and the interaction term between LI and TIC was included as the Level-2 independent variable. IIB was used as the dependent variable to construct the regression model. The results of the analysis using the cross-level model are as follows:

Level 1 Model:

$$\begin{array}{l} (\mathrm{IIB})\mathrm{ij}=\beta 0j+\beta 1j^{*}\;(\text{Genderij})+\beta 2j^{*}\;(\text{Ageij})+\\ \beta 3j^{*}\;(\text{Educationij})+\beta 4j^{*}\;(\begin{array}{l} \text{Marital}\;\\ \text{Statusij} \end{array})+\\ \beta 5j^{*}\;(\text{Employment Statusij})+\\ \beta 6j^{*}\;(\text{Years of Serviceij})+\\ \beta 7j^{*}\;(\begin{array}{l} \text{Years with Current}\;\\ \text{Supervisorij} \end{array})+\\ \beta 8j^{*}\;(\text{LMXij})+\beta 9j^{*}\;(\text{ITFLij})+\\ \beta 1{\mathrm{0j}}^{*}\;(\text{LIij})+\mathrm{rij} \end{array}$$

Level 2 Model:

$$\beta 0j=\gamma 00+\gamma 01^{*}\;(\mathrm{TIC}\;j)+\mu 0j$$

$$\begin{array}{l} \beta 1j=\gamma 10,\beta 2j=\gamma 20,\beta 3j=\gamma 30,\\ \beta 4j=\gamma 40,\beta 5j=\gamma 50,\beta 6j=\gamma 60,\\ \beta 7j=\gamma 70,\beta 8j=\gamma 80,\beta 9j=\gamma 90 \end{array}$$

$$\beta 1\mathrm{0j}=\gamma 100+\gamma 101^{*}\;(\mathrm{TIC}\;j)$$

Mixed Model:

$$\begin{array}{l} (\mathrm{IIB})\mathrm{ij}=\gamma 00+\gamma 01^{*}\;(\text{TICj})+\gamma 10^{*}\;(\text{Genderij})+\\ \gamma 20^{*}\;(\text{Ageij})+\gamma 30^{*}\;(\text{Educationij})+\\ \gamma 40^{*}\;(\text{Marital Statusij})+\\ \gamma 50^{*}\;(\text{Employment Statusij})+\\ \gamma 60^{*}\;(\text{Years of Serviceij})+\\ \gamma 70^{*}\;(\begin{array}{l} \text{Years with Current}\;\\ \text{Supervisorij} \end{array})+\\ \gamma 80^{*}\;(\text{LMXij})+\gamma 90^{*}\;(\text{ITFLij})+\\ \gamma 100^{*}\;(\text{LIij})+\gamma 101^{*}\;{\left(\mathrm{TIC}\;j\right)}^{*}\;(\text{LIij})+\\ \mu 0j+\mathrm{rij} \end{array}$$

The cross-level analysis from Model 1 to Model 4 tested the effects of different variables on IIB: In Model 1, while controlling for Level-1 variables such as gender, age, education, marital status and LMX, ITFL was included. ITFL has a significant positive impact on IIB (*β* = 0.45, *p* < 0.001). In Model 2, LI was added, demonstrating that LI has a significant positive impact on IIB (*β* = 0.15, *p* < 0.01). In Model 3, TIC was introduced, and it showed a significant positive impact on IIB (*β* = 0.19, *p* < 0.01). In Model 4, the cross-level moderating effect of TIC on the relationship between LI and IIB was tested. The interaction between LI and TIC showed a significant positive moderating effect (*β* = 0.16, *p* < 0.01).

These results confirm that the relationship between LI and IIB is strengthened when TIC is high and weakened when TIC is low. The ICC value of the model is 0.51, indicating that 51% of the variance within the model is due to team-level differences. This suggests that cross-level analysis is reasonable and necessary. The residual variance (σ^2^) and the variance of the random intercept (τ) also support the model’s fit. Therefore, hypothesis 4 is supported. The specific results are shown in Table 7.

**Table 7:** Results of cross-level hierarchical regression analysis (hypothesis 4).

Variable	IIB							
	Model 1		Model 2		Model 3		Model 4	
	*β*	*t*	*β*	*t*	*β*	*t*	*β*	*t*
Constant *γ*_00_	3.26^***^	15.47	3.31^***^	15.79	3.26^***^	15.59	3.18^***^	15.04
Gender *γ*_10_	0.01	0.42	0.01	0.42	0.01	0.39	0.01	0.47
Age *γ*_20_	0.06	1.44	0.05	1.19	0.05	1.26	0.05	1.33
Education *γ*_30_	−0.02	−0.1.01	−0.03	−1.05	−0.03	−1.20	−0.03	−1.07
Marital status *γ*_40_	0.06	1.47	0.05	1.13	0.05	1.27	0.05	1.31
Employment status *γ*_50_	0.16	1.49	0.16	1.46	0.18	1.64	0.18†	1.69
Years of service *γ*_60_	0.06	1.13	0.06	1.26	0.06	1.30	0.06	1.24
Years with current supervisor *γ*_70_	−0.04	−0.94	−0.05	−1.01	−0.04	−0.83	−0.03	−0.74
LMX *γ*_80_	0.40^***^	8.36	0.35^***^	6.91	0.28^***^	5.04	0.30^***^	5.36
ITFL *γ*_90_	0.45^***^	8.14	0.37^***^	5.82	0.37^***^	5.94	0.40^***^	6.30
LI *γ*_100_			0.15^**^	2.82	0.12^*^	2.27	0.17^**^	3.05
TIC *γ*_01_					0.19^**^	2.93	0.27^***^	4.01
LI * TIC *γ*_101_							0.16^**^	3.13
σ^2^	0.11246		0.10901		0.1088		0.11104	
τ	0.13294		0.14235		0.13553		0.11784	
ICC	0.46		0.43		0.45		0.51	

*Level 1 N = 640, Level 2 N = 160, †*p* < 0.1, *p < 0.05, **p < 0.01, ***p < 0.001; LMX, Leader-Member Exchange; ITFL, Individual-focused Transformational Leadership; LI, Leader Identification; TIC, Team Innovative Climate; IIB, Individual Innovative Behavior; σ^2^, Residual Variance; τ, Random Intercept; ICC, Variance Within the Model.

**Figure 3:**
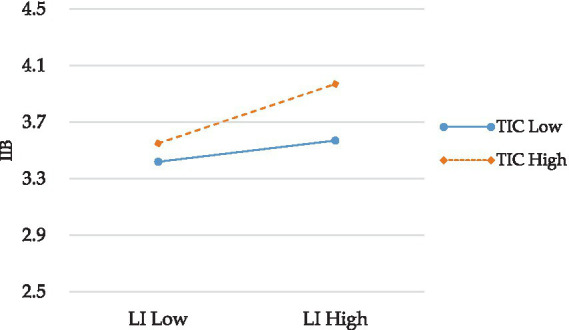
Interaction Plot of LI and TIC.

Further, a simple slope test (Figure 3) indicates that when TIC is low, the relationship between LI and IIB is weak (−1 SD; *β* = 0.01, ns), but when TIC is high, this relationship is strong (+1 SD; *β* = 0.33, *p* < 0.01). This pattern of results also supports hypothesis 4.

## 5 Conclusion, implications, and future research

### 5.1 Conclusion

To elucidate the relationship between TL and IIB, the current research pursued four specific objectives. First, it sought to explore the relationship between ITFL and IIB by distinguishing the intrinsic levels of TL. Second, it aimed to verify the mediating effect of LI in the relationship between ITFL and IIB. Third, the study examined the cross-level moderation effect of TTFL in the relationship between ITFL and LI. Lastly, it investigated the cross-level moderation effect of TIC in the relationship between LI and IIB.

There are several important findings. First, ITFL has a positive impact on IIB. Second, LI mediates the relationship between ITFL and IIB. Furthermore, TTFL exhibits a significant negative cross-level moderating effect in the relationship between ITFL and LI. Specifically, when TTFL is low, the relationship between ITFL and LI is stronger, whereas when TTFL is high, this relationship is weaker. Lastly, TIC has a significant cross-level moderation effect in the relationship between LI and IIB. This indicates that the relationship between LI and IIB is stronger when TIC is high and weaker when it is low.

### 5.2 Theoretical implications

This study makes a significant contribution to the literature on transformational leadership. TL has a significant influence on individual and team performance, however, the effect of ITFL and TTFL on individuals’ innovative behavior has not been fully explored (Kim and Hong, 2016; Sun et al., 2016).

Individuals’ motivations and abilities are crucial in driving innovative behavior (Dai et al., 2022), and team context can shape individual actions (Wang and Howell, 2012).

It is essential to distinguish between the impacts of TL at the team and individual levels. By empirically examining the effect of ITFL on IIB and the moderating effect of TTFL, the current research enhances the understanding of how the interplay between different types of TL promotes innovation.

Specifically, the findings suggest that when TTFL is present, the synergistic effect of ITFL does not emerge. This observation provides insight into the inconsistent effects of TL reported in previous research (Pang et al., 2019; Song et al., 2013; Tourish, 2013), highlighting the need for a multi-level approach to TL in future studies.

This study also extends relational identity theory by investigating the impact of TL on innovative behavior from the perspective of the leader-follower relationship. By showing how LI mediates the relationship between ITFL and IIB, this research underpins the importance of relational dynamics in fostering innovation. Additionally, the exploration of the cross-level moderating effects of team-level factors provides evidence of how team-related factors influence relationships between leaders and individuals, which in turn shapes their behaviors.

The pandemic triggered the emergence of new business routines such as remote working and virtual teams (Greimel et al., 2023; Prin and Bartels, 2020). These environmental changes call for a re-examination of existing, proven theories on the effect of leadership on members within the organization. Further, relational identity is often influenced by the specific organizational context and culture (Moriizumi, 2011; Peterson and Stewart, 2020).

Cultural norms—particularly China’s collectivist orientation—may amplify the effects of TTFL or suppress the individual autonomy encouraged by ITFL. This underscores the critical role of cultural context in leadership research. In more individualistic or egalitarian cultures, the mechanisms through which TTFL operates may differ. Future research could further explore these differentiated effects through cross-cultural comparisons to validate how leadership styles function across varying cultural contexts.

This study contributes to this discourse by empirically testing how relational identity theory applies within the collectivist culture of China, thereby expanding its applicability across various cultural contexts.

### 5.3 Practical implications

There are also practical implications of this study. The findings suggest that by enhancing ITFL within organizations, members’ innovative behavior can be effectively promoted.

ITFL primarily consists of two dimensions: individualized consideration and intellectual stimulation, which are effective in meeting individual needs and fostering creative thinking. Therefore, organizations should strengthen training programs and leadership development activities to cultivate and support transformational leadership capabilities.

Moreover, the findings of this study suggest that TIC plays an important role in moderating the relationship between LI and IIB. This implies that organizations should cultivate a cooperative and open team culture to foster a strong TIC and to enhance trust and communication among members. As TIC increases, the relationship between LI and IIB becomes stronger. Thus, organizations should implement team-building activities and programs that promote an open communication culture to create such an environment.

Lastly, the empirical results of this study indicate that the negative moderating effect of TTFL may be due to excessive vision articulation and high-performance expectations, which could burden members. Rather than emphasizing excessive vision articulation or performance expectations aimed at the entire team, it might be more effective for leaders to focus on individualized consideration and intellectual stimulation toward individual members.

When members feel valued through their leader’s individualized consideration and are encouraged to come up with new ideas and innovative behavior through intellectual stimulation, they are likely to show greater motivation and participation. By providing intellectual stimulation and support, innovative behavior can be effectively promoted. This suggests that organizations should set realistic and achievable goals and encourage members’ creativity and spontaneous innovative behavior.

In practice, it is also essential to mitigate the potential negative effects of TTFL without undermining team cohesion. Several strategies can be implemented to achieve this balance: First, organizations can design flexible leadership development programs that train team leaders to balance collective goal-setting with individualized support, helping them respond to diverse member needs while maintaining shared team direction. Second, dual-role leadership behaviors should be encouraged—leaders should deliver team vision while also demonstrating individualized consideration through regular one-on-one interactions. This approach allows members to feel both aligned with the team and personally supported. Third, team norms that encourage individual expression should be established. For example, rotating meeting facilitators or dedicating fixed time slots for members to present innovation proposals can empower individuals without diluting the team’s unity. These practices can reduce the pressure of overemphasized TTFL while reinforcing a psychologically safe and collaborative team environment.

### 5.4 Future research

While this study provides valuable insights, it is not without limitations that offer several important directions for future research. First, this study mainly focused on innovative behavior at the individual level. However, organizational innovation is influenced not only by individual innovation but also by team-level innovation. Therefore, future researchers should consider both individual- and team-level innovative behavior simultaneously (Wang and Howell, 2010). This will enable a more comprehensive approach to enhancing the innovative capacity of the entire organization.

Second, this study primarily analyzed the impact of transformational leadership on IIB through relational identity theory. However, the relationship between transformational leadership and innovative behavior needs to be explored more deeply from various theoretical perspectives. Future researchers should apply different theoretical frameworks, such as the reciprocity perspective (Wang and Howell, 2010), network perspective (Cai et al., 2013), and resource perspective (Liang et al., 2018), to gain a broader and more nuanced understanding of the effects of multilevel transformational leadership on innovative behavior.

Moreover, although the present study revealed a negative moderating effect of TTFL on the relationship between ITFL and leader identification, the underlying mechanisms were primarily explained from the lens of relational identity theory. To maintain theoretical parsimony, additional theories such as role conflict, group conformity pressure, and psychological reactance were not integrated into the main framework. Future research is encouraged to draw on these complementary perspectives to further uncover the psychological mechanisms behind the TTFL–ITFL interplay. Such theoretical integration would enrich understanding of the boundary conditions under which multilevel transformational leadership may produce differential effects.

To further enrich the exploration of leadership’s influence on innovation, subsequent directions may also involve a broader range of contextual and methodological considerations.

Third, this study set TIC as a moderating variable in the impact of multilevel transformational leadership on innovative behavior. However, there may be other moderating variables within the organization. For example, leader group prototypicality or the structural characteristics of the team could also influence innovative behavior (Diah, 2014). Future researchers should systematically explore these moderating variables to identify the conditions that can optimally maximize the effects of transformational leadership.

Fourth, this study used cross-sectional data, which, while capturing the impact of transformational leadership on innovative behavior at a specific point in time, cannot reveal the long-term dynamic changes of this impact. Innovative behavior is often an accumulative process, influenced by long-term leadership styles and organizational climate (Afsar and Umrani, 2020). Relying solely on cross-sectional data may underestimate or overestimate the effects of certain factors. Future researchers should adopt a longitudinal design, regularly tracking data to explore how transformational leadership continuously influences individual innovative behavior over time, thereby uncovering its long-term dynamic effects (Khan et al., 2020).

Additionally, we explicitly acknowledge that the cross-sectional nature of this study limits causal inference and may not fully capture the evolving leader–follower dynamics. Future research should employ longitudinal or time-lagged designs to better understand how transformational leadership exerts its influence across time. This approach would offer stronger evidence of causality and reflect the cumulative nature of innovative behavior development.

Fifth, the team-level variables in this study were limited to team-focused transformational leadership and team innovation climate, without including other control variables at the team level, such as team size, work type, or task complexity. The absence of these control variables may introduce bias into the data analysis results and affect the accuracy of the analysis. For example, larger teams may find it harder to manage innovative behavior compared to smaller teams, which could alter the actual impact of leadership styles (Cha et al., 2015). We appreciate this important limitation and have now explicitly acknowledged it. Although these variables were not included in our current model, we recommend that future research incorporate relevant team- and organizational-level control variables, such as industry context, team size, and task interdependence, to improve the robustness and generalizability of the findings. Future researchers should consider incorporating appropriate team-level control variables to enhance the accuracy of data analysis and the robustness of the results.

In remote or hybrid work contexts, transformational leadership components like individualized consideration and intellectual stimulation are especially critical, as overemphasis on unified goals (i.e., strong TTFL) may suppress personal expression; thus, leaders should focus on unlocking individual potential and building trust to foster innovation.

Lastly, this study primarily focused on transformational leadership, which, while widely recognized for its role in promoting innovative behavior, may not be the only leadership style that influences innovation. Other leadership styles may have different effects on innovative behavior. For instance, transactional leadership might motivate employees to innovate through reward mechanisms (Luo et al., 2021), while servant leadership might indirectly promote innovation by enhancing employee well-being and support systems (Zhuang and Chen, 2015). Future researchers should combine and compare various leadership styles to explore their differing impacts on innovative behavior and gain a more comprehensive leadership perspective.

## Data Availability

The datasets presented in this study can be found in online repositories. The names of the repository/repositories and accession number(s) can be found at: https://osf.io/y68gp.
